# Effectiveness of Telerehabilitation in Reducing Pain and Improving Quality of Life and Job Satisfaction Among Cardiac Sonographers with Work-Related Musculoskeletal Disorders: A Randomized Controlled Trial

**DOI:** 10.3390/jcm14186576

**Published:** 2025-09-18

**Authors:** Shibili Nuhmani, Abrar AlBakheet, Ghada AlQahtani, Rawan AlDulaijan, Madhawi AlHomiyn, Lamia Al Saikhan

**Affiliations:** 1 Department of Physical Therapy, College of Applied Medical Sciences, Imam Abdulrahman Bin Faisal University, Dammam 34212, Saudi Arabia; snuhmani@iau.edu.sa (S.N.);; 2 Department of Cardiac Technology, College of Applied Medical Sciences, Imam Abdulrahman Bin Faisal University, Dammam 34212, Saudi Arabia; 3 Department of Industrial Design, College of Design, Imam Abdulrahman Bin Faisal University, Dammam 34212, Saudi Arabia

**Keywords:** telerehabilitation, musculoskeletal pain, cardiac sonographers, occupational health, quality of life, job satisfaction

## Abstract

**Background**: Cardiac sonographers have increased risks of work-related musculoskeletal disorders (WRMDs) owing to their physically demanding work. Telerehabilitation offers a promising, accessible approach for managing WRMDs; however, evidence specific to this population remains limited. This study aimed to evaluate the effectiveness of a 6-week telerehabilitation program for musculoskeletal pain, health-related quality of life, and job satisfaction among cardiac sonographers. **Methods**: In this randomized controlled trial, cardiac sonographers with WRMDs were assigned to the telerehabilitation intervention or control group. Pain intensity (measured using the Numeric Pain Rating Scale), quality of life (assessed using the SF-36), and job satisfaction were evaluated at baseline and post-intervention. Linear mixed-effects models were used to assess differences in between-group changes over time. **Results**: Thirty-two cardiac sonographers completed the study (16 per group; mean age: 28.53 ± 4.31 years; 93.75% female). The intervention group showed significantly reduced pain intensity (*β* = −2.0; 95% CI: −3.67, −0.33; *p* = 0.019) and improved job satisfaction (*β* = 15.93; 95% CI: 3.47–28.40; *p* = 0.012) compared to controls. SF-36 subscale analysis showed significantly improved bodily pain (BP) in the intervention group (*β* = 12.81; 95% CI: 1.50–24.12; *p* = 0.026) and decreased social functioning (SF) (*β* = −11.72; 95% CI: −20.61, −2.83; *p* = 0.010); no significant between-group differences were observed for physical functioning, role physical, role emotional, vitality, general health, or mental health. **Conclusions**: This study provides evidence that telerehabilitation can effectively reduce musculoskeletal pain and enhance job satisfaction among cardiac sonographers. Although BP improved, SF declined. These findings support integrating telerehabilitation into occupational health programs, with future research needed on long-term benefits and psychosocial outcomes.

## 1. Introduction

Work-related musculoskeletal disorders (WRMDs) are one of the most prevalent musculoskeletal conditions affecting healthcare professionals involved in repetitive activities and prolonged awkward postures [1]. Cardiac sonographers are an occupational group with a high risk of developing WRMDs because of their poor ergonomic practice, maintenance of a single position for a long duration during work, and forceful grip of the ultrasound transducer [2]. Cardiac sonographers are subject to high biomechanical and ergonomic demands when performing echocardiography, which requires sustained shoulder abduction, wrist deviation, neck flexion, and prolonged static sitting [2]. Al Saikhan et al. found that most sonographers work daily for 8–9 h, and 70% of them do not have a break between scans [3]. Moreover, the repeated use of the dominant hand during scanning can result in muscular imbalance and increase the risk of developing WRMDs [4]. The most affected body parts are the shoulders [3,5,6], neck [5,7,8], hands [3,6,7], and lower back region [7,8].

WRMDs are a major cause of musculoskeletal pain in 75–90% of cardiac sonographers [6]. WRMDs are a significant occupational concern [9] that disturb the career lives of sonographers [6,10], affect their physical health, lead to absenteeism, and threaten their continuity in daily work tasks [11,12]. This decreases their ability to perform work tasks efficiently, negatively impacts their quality of life (QOL), and increases the cost of healthcare services, leading to premature career exits [4,13]. Pike et al. reported that 20% of the echocardiographers who have WRMDs complain of career-ending injuries [14]. Symptoms may appear in the first six months and affect 45% of cardiac sonographers after three years and 72% after 10 years, resulting in financial and emotional influences [15].

Despite the awareness of the challenges faced by this occupational group, studies regarding the implementation and effectiveness of a structured rehabilitation program that specifically targets cardiac sonographers are limited. A traditional rehabilitation program may be beneficial in improving this condition, but its benefits are limited by challenges such as accessibility, time constraints, and a shortage of therapists, which may ultimately lead to poor adherence to the program and discontinuation of treatment. Telerehabilitation (TR) is an acceptable alternative to traditional rehabilitation for managing various WRMDs [16,17].

TR is defined as providing virtual rehabilitation to patients utilizing telecommunications through different technologies such as phone and video calling, virtual reality [18,19], mobile applications, and exercise programs based on the Internet [20,21]. Over the past decade, TR has emerged as an effective method for managing various musculoskeletal and neurological disorders, enabling rehabilitation professionals to assess and manage patients remotely without requiring hospital attendance. Studies have demonstrated that TR is a safe, effective, and accessible approach for managing musculoskeletal and work-related disorders, offering benefits such as reduced pain, improved QOL, cost efficiency, and greater flexibility in care delivery [16]. The advantages of TR include easy accessibility to high-quality health services, cost reduction, regular contact with a therapist, and flexibility in the rehabilitation program, resulting in improved health outcomes [16], decreased musculoskeletal pain, and improved QOL [22]. Amin et al. found that TR was effective in managing musculoskeletal disorders and is an acceptable alternative method for patient consultation instead of face-to-face visits in the hospital [23]. In addition, Baroni et al. demonstrated that TR is a safe and effective way to manage WRMDs [24]. Similarly, Cottrell et al. found that TR is an effective method that provides easy accessibility to rehabilitation services, compared to the traditional approach [17]. However, its application, specifically for addressing WRMDs in cardiac sonographers, has not been sufficiently studied.

Due to the unique occupational risk and job pattern of cardiac sonographers, a tailored rehabilitation intervention program focusing on the self-management of pain, postural correction, ergonomic education, and strengthening of the core and scapular muscles can be beneficial in relieving pain and other WRMD-related symptoms. TR-delivered programs can be personalized, interactive, and performed without affecting daily work-related activities. Recent evidence suggests that healthcare professionals are receptive to TR and other technology-based rehabilitation strategies that offer flexibility and continuity. To the best of our knowledge, no interventional studies have investigated the effectiveness of TR specifically in cardiac sonographers. Therefore, the objective of this study was to investigate the effectiveness of TR in reducing pain and improving the QOL and work satisfaction in cardiac sonographers with WRMDs.

This randomized controlled trial (RCT) aims to provide actionable insights to inform occupational health policy, support routine integration of TR in daily practice. If effective, the program could enhance well-being and QOL and support job satisfaction, encouraging rehabilitation professionals and cardiac sonographers to adopt TR-based exercise for the prevention and management of WRMSDs.

## 2. Materials and Methods

### 2.1. Study Design

This study was a single-blind two-arm RCT with a pre-test and post-test design registered with ClinicalTrials.gov (registration number: NCT06842485; date: 25 February 2025). The participants were blinded to the group allocation. This study was approved by the Institutional Review Board (IRB) of Imam Abdulrahman bin Faisal University (IRB number: IRB-2025-03-0129, Approval Date: 16 February 2025), and all procedures adhered to the Declaration of Helsinki. Prior to participation, the study aims and procedures were explained to the participants, and written informed consent was obtained. This study is reported in accordance with the CONSORT 2010; the completed CONSORT 2010 checklist is provided as Supplementary File S1.

### 2.2. Sample Size Calculation

The sample size was calculated using the Harvard University Sample Size Calculator (available at https://hedwig.mgh.harvard.edu/sample_size/ accessed on 8 January 2025) to achieve 80% power at a 0.05 significance level (alpha) to detect a minimal clinically important difference (MCID) of 1.1 points on the Numeric Pain Rating Scale (NPRS), based on previously published data [25]. Thirty participants were determined to be sufficient for this two-group parallel design study, with equal allocation to each treatment group. To accommodate a potential 10% dropout rate, the final target sample size was increased to 33 participants. The final number of enrolled participants was 32, with 16 participants in each group.

### 2.3. Participants

A total of 32 cardiac sonographers who are working in various government or private hospitals in Saudi Arabia participated in this study. All recruited cardiac sonographers performed adult and/or pediatric echocardiography. The inclusion criteria were adults (age ≥ 18 years); currently employed as a cardiac sonographer with active scanning duties; self-reported WRMD symptoms in neck, shoulder, upper or lower back, or wrist/hand region; and willingness to attend video based supervised TR program. Exclusion criteria were systemic diseases likely to limit safe participation; use of cardiac medications or painkillers; other musculoskeletal disorders or injuries unrelated to WRMDs; recent surgery that may affect their ability to participate in the exercise program; other contraindications to participate in the exercise program; and pregnancy.

### 2.4. Randomization

Eligible participants were divided into two groups (control and intervention groups) at an allocation ratio of 1:1 using the RAND function in Microsoft Excel. The allocation was concealed in a non-transparent envelope and disclosed only at the time of the intervention. Participant enrollment, randomization, and allocation concealment were performed by an independent researcher who was not affiliated with this study.

### 2.5. Procedure

An invitation to participate in the study was advertised through social media and noticeboards in different private and governmental hospitals. Initial screening of the participants was performed by a qualified physical therapist based on the inclusion and exclusion criteria. The demographic and anthropometric data of the participants were collected during this session using screening and data collection forms [26,27,28].

### 2.6. Intervention

Participants in the control group received a structured patient education program provided by a qualified physiotherapist, which lasted for 30 min. The educational program was sent to participants via email and WhatsApp. The program focused on postural and ergonomic strategies to reduce the risk of WRMDs. The participants were instructed to maintain a good posture during the patient examination by adjusting the height of the chair and examination bed. They were advised to support the back of the chair and use back and seat cushions for more support. To avoid a static position for a prolonged period of time, they were encouraged to change their position every 45 min; take short breaks; change procedures, tasks, and activities whenever possible; and look at faraway objects to decrease stress on the eyes [10]. The participants were advised to adjust their hand position to grasp the transducer and avoid wrist flexion during the patient examination. The participants were also instructed to avoid flexing and abducting the right shoulder by more than 90° during the patient examination, to decrease the chance of right shoulder and neck pain, and avoid cervical extension and rotation during the patient examination [29].

Tailored TR and patient education programs were provided to the intervention groups. The patient education program was identical to that of the control group. The TR program involved active virtual patient-therapist participation, with less direct therapist-patient interaction compared to traditional treatment methods [30]. The TR program was developed based on previous WRMDs prevention programs, which demonstrated the effectiveness of therapeutic exercises based on TR in reducing musculoskeletal pain [18], the rate of absenteeism, and improving QOL and productivity for employees who complain of neck, shoulder, and low back pain [22]. The TR phase provided therapeutic exercises for the patient’s specific condition and complaints, which assisted in reducing pain and improving work productivity [22]. The program included stretching and isometric strengthening exercises for cervical muscles, strengthening exercises for the shoulder muscles, stretching exercises for the wrist, McKenzie extension exercises for the low back, and core stability exercises. The 30 min sessions were virtual via Zoom Video Communications (Inc., San Joes, CA, USA) and lasted six weeks, three days per week. Table 1 lists the program details. The privacy of participants was considered during TR sessions, and good communication was ensured to build trust with the participants and show empathy [24].

### 2.7. Outcome Measures

The primary outcome measure was pain intensity measured using the NPRS. The NPRS is a valid and reliable tool for measuring pain intensity that uses an 11-point scale ranging from 0 (no pain) to 10 (severe pain). The participants selected a single number that best described the intensity of their pain [26].

Additional outcome measures included the QOL and job satisfaction. The QOL was measured using the SF-36 questionnaire. The SF-36 demonstrates strong reliability and validity for measuring health perception and QOL across a broad population and accurately identifying health variations across demographic and health status categories. The SF-36 questionnaire consists of 36 items organized into eight dimensions: physical functioning (PF), social functioning (SF), role limitations due to physical and emotional problems (RP and RE, respectively), mental health (MH), vitality (VT), bodily pain (BP), and general health perception (GH). The questionnaire uses three- or six-point rating scales for patient responses in six dimensions, to detect both positive and negative health states. This approach enables a comprehensive evaluation of health. The eight dimensions were coded, summed, and transformed into a standardized scale ranging from 0 to 100. This process provided a score profile, allowing the detection of intermediate health states and low levels of ill health [27].

The Job Satisfaction Survey (JSS) was used to assess work satisfaction. The JSS used a five-point Likert scale designed for healthcare providers. It comprised 49 items related to job satisfaction and seven determinants: privileges attached to the job, interpersonal relations and cooperation, working environment, patient relationships, organizational facilities, career development, human resource issues, and cultural context. The results were calculated and interpreted based on this scale, with each item assigned a score. The scale has demonstrated high reliability and validity. The interpretation of the satisfaction level was based on the midpoint of the scale, which was 2.5. Scores > 2.5 indicated job satisfaction, whereas scores < 2.5 indicated job dissatisfaction [28].

### 2.8. Statistical Analysis

All analyses were performed using Stata (version 15.0; StataCorp, College Station, TX, USA). Continuous variables are presented as means ± standard deviations or medians with interquartile ranges, and categorical variables are presented as frequencies and percentages. Baseline comparisons between groups were performed using the independent samples *t*-test or Mann–Whitney U test for continuous variables and the chi-square or Fisher’s exact test for categorical variables, as appropriate.

To evaluate the intervention effects over time, separate linear mixed-effects models were fitted for each outcome: pain intensity, overall health-related QOL (SF-36 total score), and work satisfaction. Each model included fixed effects for group (intervention vs. control), time (pre- and post-intervention), and their interactions with random intercepts to account for repeated measures. This approach provided robust estimates of whether the change in outcomes over time differed between groups.

Linear mixed-effects models were selected over traditional two-way ANOVA and ANCOVA because of their superior ability to account for the correlated nature of repeated measures data [30,31]. Unlike two-way ANOVA and ANCOVA, which assume the independence of observations and often require complete data across all time points, mixed models accommodate intra-individual correlations by including random effects, allow for unbalanced or missing data without excluding entire cases, and provide greater flexibility in modeling time as a factor or covariate [31,32]. This makes them particularly appropriate for longitudinal studies with repeated observations per subject.

The primary hypothesis testing focused on the group × time interaction term, assessing whether changes over time differed significantly between the groups. The model assumptions were checked using residual analysis and diagnostic plots to ensure validity. Two-sided *p*-values < 0.05 were considered statistically significant.

## 3. Results

### 3.1. Baseline Characteristics of Cardiac Sonographers

**Figure 1:**
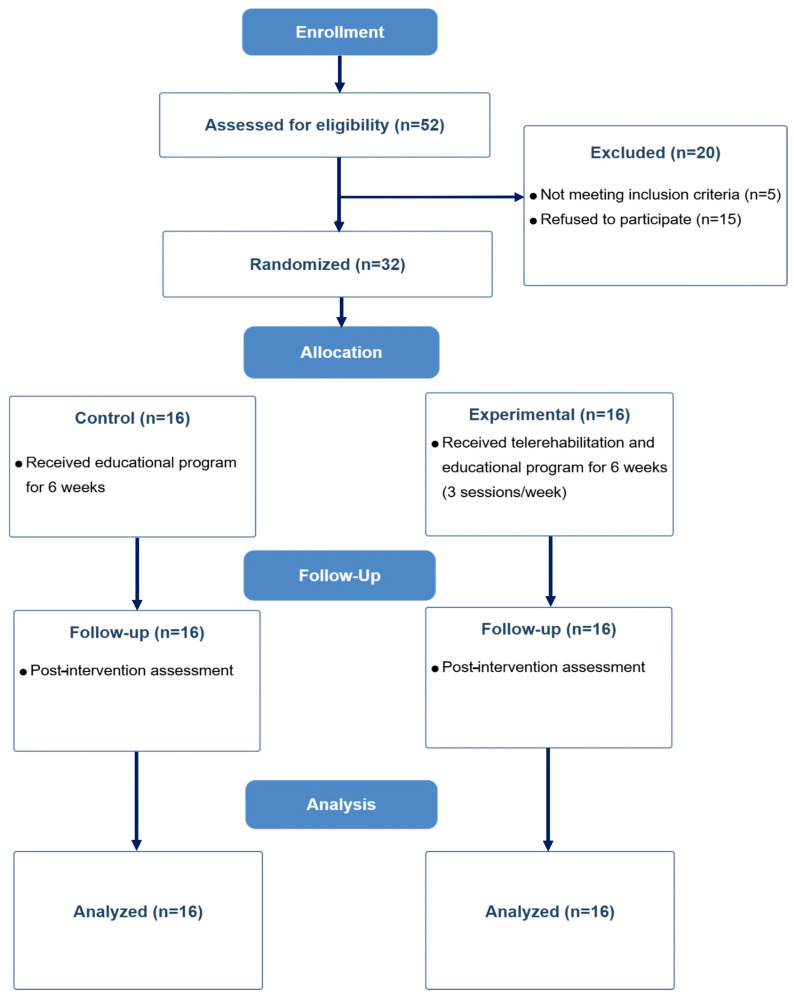
Consolidated standards of reporting trials (CONSORT) flowchart showing the numbers of participants assessed, recruited, randomized, and analyzed during the study.

In total, 52 cardiac sonographers were screened for eligibility. Of them, 20 were excluded (Figure 1). The remaining 32 cardiac sonographers met the inclusion criteria and were equally randomized into the intervention (*n* = 16) and control (*n* = 16) groups. All participants completed the study and were included in the final analysis. The baseline sociodemographic and clinical characteristics are summarized in Table 2. The two groups were comparable across all variables, except for age, where the control group was significantly younger than the intervention group (mean age: 26.87 ± 0.79 vs. 30.18 ± 1.18 years; *p* = 0.027).

The baseline occupational characteristics of the cardiac sonographers are summarized in Table 3. No significant differences were found between the intervention and control groups for most measured variables (*p* > 0.05). Controls reported more exam/task rotations than the intervention group (87.5% vs. 50.0%), although this difference was not significant (*p* = 0.054).

### 3.2. Primary Outcome (Pain Intensity [NPRS])

**Figure 2:**
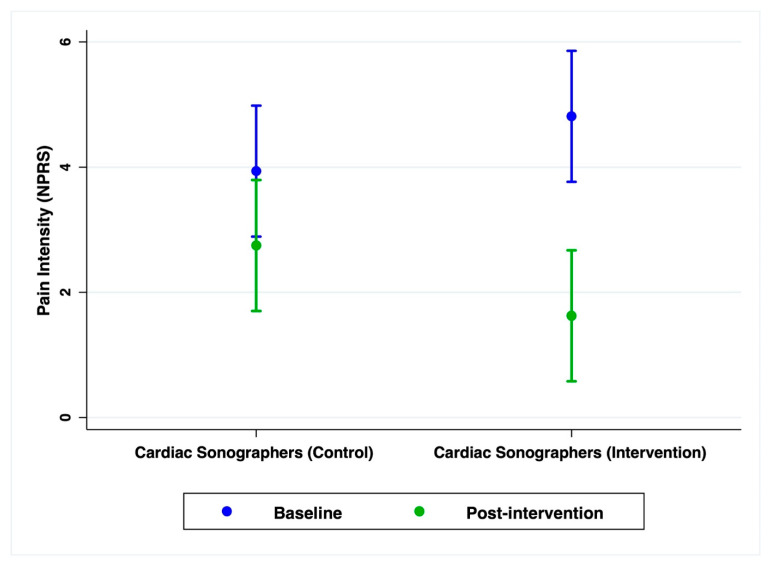
Estimated marginal mean pain intensity (NPRS score) across time by group. The intervention group showed a significantly greater reduction in pain scores over time compared to the control group (*p* = 0.019). Error bars represent 95% confidence intervals.

Over the 6-week intervention period, participants in the intervention group experienced a significant reduction in pain intensity compared to the control group (*β* = −2.0, 95% confidence interval [CI: −3.67, −0.33], *p* = 0.019), indicating a more pronounced decline in NPRS scores among those receiving the intervention (Table 4). Adjusted mean NPRS scores in the intervention group decreased from 4.81 (95% CI [3.77, 5.86]) at baseline to 1.63 (95% CI [0.58, 2.67]) post-intervention. In contrast, the control group showed a more modest reduction, from 3.94 (95% CI [2.89, 4.98]) to 2.75 (95% CI [1.70, 3.80]) (Figure 2, Table 4).

### 3.3. Secondary Outcomes (QOL and Job Satisfaction)

#### 3.3.1. Job Satisfaction

**Figure 3:**
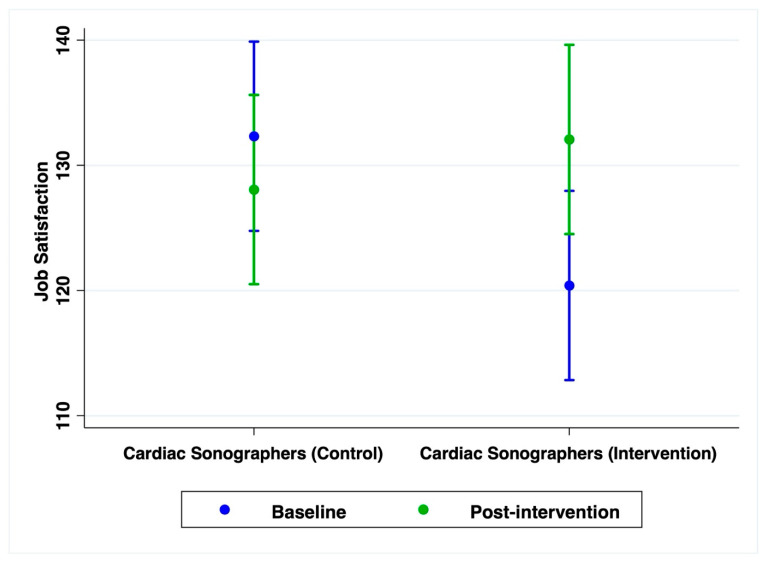
Estimated marginal means of job satisfaction across time by group. The intervention group showed a significantly greater improvement in job satisfaction from baseline to post-intervention compared to the control group (*p* = 0.012). Error bars represent 95% confidence intervals.

Over the 6-week intervention period, participants in the intervention group showed a significantly greater improvement in job satisfaction from baseline to post-intervention compared to the control group (*β* = 15.93, 95% CI [3.47, 28.40], *p* = 0.012) (Table 4). The adjusted mean job satisfaction score in the intervention group increased from 120.4 (95% CI [112.84, 127.96]) at baseline to 132.07 (95% CI [124.51, 139.62]) post-intervention. In contrast, the control group showed a slight decrease over the same period, from 132.33 (95% CI [124.78, 139.89]) to 128.07 (95% CI [120.51, 135.62]) (Figure 3, Table 4).

#### 3.3.2. QOL

No significant differences were observed between the intervention and control groups in the trajectories of health-related QOL over time (*p* = 0.490) (Table 4). Adjusted mean scores remained stable with overlapping 95% CI between groups at all assessment points, suggesting no differential effect of the intervention on this secondary outcome.

No significant differences were observed between the intervention and control groups in the trajectories of SF-36 subscale outcomes over time for PF, RP, RE, VT, GH, or MH. However, the intervention group showed a significant improvement in BP compared to the control group (*β* = 12.81; 95% CI [1.50, 24.12], *p* = 0.026). Conversely, SF decreased in the intervention group relative to controls (β = −11.72; 95% CI [−20.61, −2.83], *p* = 0.010) (Table 5).

## 4. Discussion

This study investigated the effectiveness of a 6-week tailored TR program in reducing work-related musculoskeletal pain and improving job satisfaction and QOL among cardiac sonographers, a workforce particularly vulnerable to occupational strain due to the physical demands of their work. To our knowledge, this is the first study to assess the efficacy of a TR intervention in this specific population. The results indicated that the TR group experienced a significant reduction in pain intensity and an improvement in job satisfaction. Although the overall SF-36 score, which assessed QOL, did not differ significantly, subscale analysis showed a reduction in the BP domain, and a reduction in SF in the intervention group.

Our findings are consistent with a growing body of evidence supporting TR interventions in the management of musculoskeletal disorders. The target population in our study, cardiac sonographers, is a high-risk occupational group prone to developing WRMDs because of their awkward posture during practice, repetitive upper limb movements, and forceful grip of the ultrasound transducer [2]. Previous studies have reported a high prevalence of WRMDs among this population, especially affecting the shoulder, neck, and lower back regions, which can lead to poor job performance and negatively impact QOL [3,5,6].

Several clinical trials and systematic reviews have investigated the efficacy of TR interventions in various musculoskeletal conditions [18,33,34,35,36]. A meta-analysis by Suso-Martí et al. [37] reported that TR showed positive clinical results comparable with the traditional face-to-face rehabilitation approaches in improving physical function and health-related QOL. Similarly, an RCT by Azma et al. [36] found no significant difference between traditional rehabilitation and TR in relieving symptoms and improving clinical outcomes in patients diagnosed with osteoarthritis. These findings support the argument that a TR program may be an effective method for managing musculoskeletal disorders when delivered appropriately.

A recent systematic review by Amin et al. [23] found that patient satisfaction with a TR program is influenced by the perceived performance, technological quality, and therapeutic relationships. These core areas were carefully preserved in our TR program through the real-time supervision by a qualified physical therapist and structured communication between the therapist and participants. Additionally, Cottrell and Russell [38] highlighted that patient adherence to exercise and rehabilitation protocols is better with TR interventions, which is a critical factor in recovery and pain reduction in rehabilitation. This may explain the positive results observed in this study. The TR protocol used in our study was specifically designed to address the occupational demands and anatomical risk areas of the targeted population. The therapeutic exercises targeted the cervical, shoulder, wrist, and lower back regions, which previous studies have reported as high-risk areas for musculoskeletal injuries among cardiac sonographers.

The pain intensity was significantly reduced in the TR group by an average of 3.18 points on the NPRS (from 4.81 at baseline to 1.63 post-intervention), which exceeded the MCID threshold of the NPRS. This indicates the therapeutic importance of TR programs in managing musculoskeletal disorders. Comparable pain reduction has also been reported in other clinical trials with TR intervention, highlighting the growing recognition of TR programs as a viable alternative to conventional face-to-face rehabilitation methods [39]. For instance, a mean reduction of 2.9 points on the NPRS was reported in a single-arm prospective clinical trial following a structured TR intervention in patients with musculoskeletal disorders [39].

Our study also reported a significant improvement in job satisfaction in the TR group. This finding is particularly important in the healthcare sector, where WRMDs can lower confidence, contribute to burnout, and, in some cases, lead to early retirement. While direct trials using JSS tools are limited, various studies have reported improvements in the determinants of job satisfaction following TR programs [23,40]. In a prospective cohort of 5032 employees, a 12-week multimodal digital musculoskeletal rehabilitation program demonstrated meaningful improvements in work productivity and a reduction in overall productivity impairment [40]. In addition, a systematic review by Amin et al. (2022) reported higher satisfaction with TR programs among both patients and rehabilitation professionals [23]. Job satisfaction is an attitudinal outcome, reflecting participants’ feelings about their work. Therefore, the improvement in the JSS scale observed in our study indicates a positive shift in work attitude. However, it does not directly measure changes in productivity, absenteeism, or retention.

Although there was no significant difference in the SF-36 scores between the groups following the interventions, subscale analysis revealed a reduction in BP and a decline in SF in the TR group. The decline in the BP domain aligns with the reduction in pain intensity measured by the NPRS, reflecting improved PF in the target population following the intervention. Simultaneously, a reduction in the SF domain was observed, suggesting that the home-based nature of TR may have a negative impact on psychosocial functions. This may be due to the virtual nature of the TR program, which limits the social interaction and peer support of traditional face-to-face rehabilitation. In addition, the program consisted of three sessions per week, and each session lasted for more than 30 min, which might have affected the participants’ other social and household responsibilities. The TR program in our study used synchronous, one-to-one sessions with the therapist contact only. Social outcomes appear to be sensitive to the mode of delivery and program design. Group-based synchronous cardiac TR often reports improvements in QOL, likely due to the inclusion of real-time interaction and built in social support [41]. At the same time, evidence for improvements specifically in the SF-36 SF domain with predominantly in asynchronous models is limited [42]. Importantly, the SF-36 SF scale measures the extent to which health problems interfere with everyday social activities; therefore, even effective rehabilitation may unintentionally reduce the time availability for family, community, or workplace interactions. Future programs may consider integrating peer interaction, group-based modules, or other methods to preserve and enhance the social connections of the participants, potentially mitigating this effect.

Our study had several strengths that add confidence to the findings. The RCT design and absence of participant attrition throughout the 6-week rehabilitation program enhanced the reliability of the results. In addition, the use of valid and reliable outcome measures, such as the NPRS, JSS, and SF-36, improved the accuracy of the outcome measures. We used a linear mixed-effects model for statistical analysis, which is a robust method for determining changes over time.

However, this study had several limitations. First, some participants demonstrated low responsiveness and inconsistent involvement, which may have reduced adherence to the TR program; to address this, we sent scheduled reminders to encourage attendance. Second, the sample size was modest, which limited statistical precision, prevented subgroup analyses, and reduced the generalizability of the findings. Third, the intervention and follow-up periods were relatively short, making it unclear whether improvements in pain and job satisfaction will be sustained long-term. Further research with a longer duration and extended follow-up is warranted to establish the long-term effectiveness of TR programs. Fourth, the TR group necessarily received greater therapist contact than the control, so non-specific attention effects cannot be entirely ruled out as partial contributors to between-group differences. Fifth, our sample consisted of cardiac sonographers working in Saudi Arabia. Therefore, the result may not be generalizable to other cultural settings, healthcare systems, or imaging professions. Finally, baseline differences in age between the intervention and control groups may have introduced confounding. Considering these limitations, the results of this study should be interpreted with caution.

## 5. Conclusions

This study provides the first evidence that TR is an effective approach for reducing musculoskeletal pain and enhancing job satisfaction among cardiac sonographers. Although an improvement in BP was evident, SF declined. Future studies should explore the long-term benefits of TR programs and strategies to improve psychosocial aspects, in addition to physical recovery.

## Figures and Tables

**Table 1:** Telerehabilitation program provided to the experimental group.

Body Region	Exercise	Benefit	Number of Repetitions
Neck	Active stretching exercises for cervical flexors and lateral flexors	Improve cervical flexibility Increase length of tight cervical muscles Reduce neck pain	30–60 s hold, 2–4 repetitions
	Isometric strengthening exercises for cervical muscles	Reduce neck pain Increase the strength of weak cervical muscles	10 s hold, 10 repetitions 1 set
Shoulder	Shoulder flexion exercise from a standing position	Improve mobility Improve blood circulation Reduce pain Enhance shoulder joint flexibility	Isotonic: 15 repetitions, 3 sets Isometric: 6 sets, 30 s/set with 2 min rest
	Shoulder external rotation exercise from a supine position	Improve mobility Improve blood circulation Reduce pain Enhance shoulder joint flexibility	Isotonic: 15 repetitions, 3 sets Isometric: 6 sets, 30 s/set with 2 min rest
Wrist	Active stretching exercise for wrist flexors	Improve thumb mobility Reduce pain Enhance wrist flexibility	30–60 s hold, 2–4 repetitions
	Active stretching exercise for the thenar muscle	Improve thumb mobility Reduce pain Enhance wrist flexibility	30–60 s hold, 2–4 repetitions
Low back	McKenzie extension exercises from prone and standing positions	Improve lower back mobility Decrease LBP Increase lumbar flexibility	10 repetitions, 10 s hold, 1 set
	Core stability exercise (Bridge exercise)	Decrease LBP Increase core muscle strength	10 repetitions, 10 s hold, 1 set

**Table 2:** Baseline characteristics of cardiac sonographers.

Variable	Control Group (*n* = 16)	Intervention Group (*n* = 16)	*p*-Value
Age, years	26.88 [25.18–28.57]	30.19 [27.66–32.71]	0.027
Female, *n* (%)	15 (93.75)	15 (93.75)	1.000
Height, cm	160.25 [157.07–163.43]	160.25 [157.05–163.45]	1.000
Weight, kg	60.69 [54.45–66.93]	63.94 [56.10–71.77]	0.494
Body mass index, kg/m^2^	23.30 [21.41–25.19]	23.22 [19.47–26.98]	0.968
Level of education, *n* (%)			1.000
Bachelor	14 (87.5)	14 (87.5)	
Masters	0 (0)	1 (6.25)	
Diploma	2 (12.5)	1 (6.25)	
Cardiac sonography, *n* (%)			1.000
Adult	15 (93.75)	15 (93.75)	
Pediatric	1 (6.25)	1 (6.25)	
Place of employment, *n* (%)			0.716
Government hospital	9 (56.25)	11 (68.75)	
Private hospital	6 (37.50)	5 (31.25)	
Private outpatient clinic	1 (6.25)	0 (0)	

Data are expressed as count (%) or mean [95% CI].

**Table 3:** Baseline occupational characteristics of cardiac sonographers.

Variable, *n* (%)	Control Group (*n* = 16)	Intervention Group (*n* = 16)	*p*-Value
Years of experience (years)			
<1	4 (25.00)	3 (18.75)	0.449
1–5	8 (50.00)	5 (31.25)	
>5–10	4 (25.00)	6 (37.50)	
>10–15	0 (0.00)	2 (12.50)	
Primary scanning hand			
Right	13 (81.25)	11 (68.75)	0.394
Left	1 (6.25)	0 (0.00)	
Both	2 (12.50)	5 (31.25)	
Overnight calls	6 (37.50)	10 (62.50)	0.157
Work weekends	7 (43.75)	7 (43.75)	1.000
Hours spent scanning per day			
≤4	1 (6.25)	2 (12.50)	1.000
4–6	6 (37.50)	6 (37.50)	
6–8	8 (50.00)	8 (50.00)	
≥8	1 (6.25)	0 (0.00)	
Exam/task rotation	14 (87.50)	8 (50.00)	0.054
Breaks between booked scans			
Yes	1 (6.25)	3 (18.75)	0.702
No	8 (50.00)	6 (37.50)	
Sometimes	7 (43.75)	7 (43.75)	
Assigned scans per day			
≤5	1 (6.25)	1 (6.25)	0.894
5–7	7 (43.75)	6 (37.50)	
7–9	6 (37.50)	5 (31.25)	
≥10	2 (12.50)	4 (25.00)	
Average time per scan (min)			
≤30	5 (31.25)	7 (43.75)	0.560
30–45	8 (50.00)	8 (50.00)	
45–60	3 (9.38)	1 (6.25)	
Working hours per day			
7	0 (0.00)	1 (6.25)	0.685
8	13 (81.25)	11 (68.75)	
9	3 (18.75)	4 (25.00)	

Data are expressed as count (%).

**Table 4:** Estimated means and between-group differences over time for study outcomes.

Outcome	Time Point	Control Group Adjusted Mean (95% CI)	Intervention Group Adjusted Mean (95% CI)	Group × Time Interaction (*β*, 95% CI)	*p*-Value
Pain (NPRS)	Baseline	3.94 (2.89, 4.98)	4.81 (3.77, 5.86)	-	-
	Post-intervention	2.75 (1.70, 3.80)	1.63 (0.58, 2.67)	−2.0 (−3.67, −0.33)	0.019
Job Satisfaction	Baseline	132.33 (124.78, 139.89)	120.4 (112.84, 127.96)	-	-
	Post-intervention	128.07 (120.51, 135.62)	132.07 (124.51, 139.62)	15.93 (3.47, 28.40)	0.012
SF-36 Total	Baseline	95.68 (91.16, 100.21)	97.31 (92.79, 101.84)	-	-
	Post-intervention	98.75 (94.23, 103.27)	98.00 (93.48, 102.52)	−2.38 (−9.11, 4.36)	0.490

Adjusted means and interaction effects were estimated using linear mixed-effects models, including fixed effects for group, time, and interactions with random intercepts for participants. The group × time interaction term represents the differential change in the outcome over time between the groups. *p*-values indicate statistically significant differences (*p* < 0.05).

**Table 5:** Estimated means and between-group differences over time for SF-36 subscale outcomes.

Outcome	Time Point	Control Group Adjusted Mean (95% CI)	Intervention Group Adjusted Mean (95% CI)	Group × Time Interaction (*β*, 95% CI)	*p*-Value
PF	Baseline	81.25 (71.11, 91.39)	80.67 (70.19, 91.14)	-	-
	Post-intervention	84.69 (74.54, 94.83)	79.67 (69.19, 90.14)	−4.44 (−15.86, 6.98)	0.446
RP	Baseline	65.63 (49.15, 82.10)	68.75 (52.28, 85.22)	-	-
	Post-intervention	75.0 (58.53, 91.47)	81.25 (64.78, 97.72)	3.13 (−24.90, 31.15)	0.827
RE	Baseline	52.08 (33.29, 70.88)	56.25 (37.45, 75.05)	-	-
	Post-intervention	72.92 (54.12, 91.71)	68.75 (49.95, 87.55)	−8.33 (−38.20, 21.53)	0.584
VT	Baseline	46.56 (38.56, 54.57)	40.31 (32.31, 48.32)	-	-
	Post-intervention	51.25 (43.24, 59.26)	52.81 (44.81, 60.82)	7.81 (−4.33, 19.95)	0.207
BP	Baseline	64.34 (56.17, 72.58)	56.72 (48.51, 64.92)	-	-
	Post-intervention	67.34 (59.14, 75.55)	72.5 (64.29, 80.71)	12.81 (1.50, 24.12)	0.026
GH	Baseline	62.81 (53.43, 72.19)	54.34 (45.0, 63.75)	-	-
	Post-intervention	68.13 (58.75, 77.50)	60.94 (51.56, 70.32)	1.25 (−8.67, 11.17)	0.805
SF	Baseline	52.34 (47.25, 57.44)	57.03 (51.93, 62.13)	-	-
	Post-intervention	57.03 (51.93, 62.13)	50.0 (44.90, 55.10)	−11.72 (−20.61, −2.83)	0.010
MH	Baseline	59.06 (50.35, 67.78)	53.25 (44.53, 61.97)	-	-
	Post-intervention	64.5 (55.78, 73.22)	64.5 (55.78, 73.21)	5.81 (−5.44, 17.07)	0.311

Adjusted means and interaction effects were estimated using linear mixed-effects models, including fixed effects for group, time, and interactions with random intercepts for participants. The group × time interaction term represents the differential change in the outcome over time between the groups. *p*-values indicate statistically significant differences (*p* < 0.05). Abbreviations: PF, physical functioning; RP, role functioning/physical; RE, role functioning/emotional; VT, vitality; BP, bodily pain; GH, general health perception; SF, social functioning; MH, mental health.

## Data Availability

The original contributions presented in the study are included in the article/Supplementary Materials, further inquiries can be directed to the corresponding author.

## Supplementary Materials

The following supporting information can be downloaded at: https://www.mdpi.com/article/10.3390/jcm14186576/s1, File S1: CONSORT 2010 checklist of information to include when reporting a randomised trial.

## Abbreviations

The following abbreviations are used in this manuscript:

WRMD	Work-related musculoskeletal disorders
QOL	Quality of life
TR	Telerehabilitation
IRB	Institutional review board
MCID	Minimal clinically important difference
NPRS	Numerica Pain Rating Scale
JSS	Job Satisfaction Survey
PF	Physical functioning
RP	Role physical
RE	Role emotional
VT	Vitality
GH	General health
MH	Mental health
BP	Bodily pain
SF	Social functioning
RCT	Randomized controlled trial
CI	Confidence interval
